# Drought and heat stress on cotton genotypes suggested agro-physiological and biochemical features for climate resilience

**DOI:** 10.3389/fpls.2023.1265700

**Published:** 2023-10-30

**Authors:** Muhammad Mubashar Zafar, Waqas Shafqat Chattha, Azeem Iqbal Khan, Saba Zafar, Mishal Subhan, Huma Saleem, Arfan Ali, Aqsa Ijaz, Zunaira Anwar, Fei Qiao, Amir Shakeel, Mahmoud F. Seleiman, Daniel O. Wasonga, Aqsa Parvaiz, Abdul Razzaq, Jiang Xuefei

**Affiliations:** ^1^ Sanya Institute of Breeding and Multiplication, Hainan University, Sanya, China; ^2^ School of Tropical Agriculture and Forestry, Hainan University, Danzhou, China; ^3^ State Key Laboratory of Cotton Biology, Key Laboratory of Biological and Genetic Breeding of Cotton, The Ministry of Agriculture, Institute of Cotton Research, Chinese Academy of Agricultural Science, Anyang, Henan, China; ^4^ Department of Plant Breeding and Genetics, University of Agriculture Faisalabad, Faisalabad, Pakistan; ^5^ Department of Biochemistry and Biotechnology, The Women University Multan, Multan, Pakistan; ^6^ Department of Microbiology and Molecular genetics, The Women University Multan, Multan, Pakistan; ^7^ FB Genetics, Four Brothers Group, Lahore, Pakistan; ^8^ Nuclear Institute for Agriculture and Biology College (NIAB-C), Pakistan Institute of Engineering and Applied Sciences (PIEAS), Nilore, Islamabad, Pakistan; ^9^ Plant Production Department, College of Food and Agriculture Sciences, King Saud University, Riyadh, Saudi Arabia; ^10^ Department of Crop Sciences, University of Illinois Urbana-Champaign, Urbana, IL, United States; ^11^ Institute of Molecular Biology and Biotechnology, The University of Lahore, Lahore, Pakistan

**Keywords:** climate change, heat stress, drought stress, abiotic stress, physiology

## Abstract

This study aimed to investigate the impact of individual drought, heat, and combined drought and heat stress on twelve cotton genotypes, including eight tolerant and four susceptible genotypes. A field experiment was carried out by employing a randomized complete block split-plot design, with treatments (control, drought, heat, drought + heat), and cotton genotypes assigned to the main plots and sub-plots respectively. The results showed that the combined stress had a more severe impact on the yield and fiber quality of cotton genotypes compared to individual stresses. Among the studied genotypes, FB-Shaheen, FH-207, MNH-886, and White Gold exhibited superior performance in regard to agronomic and fiber quality characters under combined stress environments. Physiological parameters, including transpiration rate, stomatal conductance, relative water contents, and photosynthetic rate, were significantly reduced under combined stress. However, specific genotypes, MNH-886, FH-207, White Gold, and FB-Shaheen, demonstrated better maintenance of these parameters, indicating their enhanced tolerance to the combined stress. Furthermore, the accumulation of reactive oxygen species was more pronounced under combined stress compared to individual stressors. Tolerant genotypes showed lower levels of H_2_O_2_ and MDA accumulation, while susceptible genotypes exhibited higher levels of oxidative damage. Antioxidant enzyme activities, such as superoxide dismutase, peroxidase, and catalase, increased under combined stress, with tolerant genotypes displaying higher enzyme activities. Conversely, susceptible genotypes (AA-703, KZ 191, IR-6, and S-15) demonstrated lower increases in enzymatic activities under combined stress conditions. Biochemical traits, including proline, total phenolic content, flavonoids, and ascorbic acid, exhibited higher levels in resistant genotypes under combined stress, while sensitive genotypes displayed decreased levels of these traits. Additionally, chlorophyll a & b, and carotenoid levels were notably decreased under combined stress, with tolerant genotypes experiencing a lesser decrease compared to susceptible genotypes.

## 1 Introduction

The continuous and persistent shift in environmental conditions poses an ongoing threat to the productivity and sustainability of major field crops, thereby impacting their developmental behavior and ability to withstand adverse environmental conditions (Noman and Azhar, 2023; Yuan et al., 2023). Climate change is exacerbated by the degradation of croplands caused by desertification, salinization, urbanization, and unregulated population growth (Zhu et al., 2023). The productivity of crop plants is influenced by various climatic factors, including drought, heat, precipitation, humidity, and sunshine hours (Zafar et al., 2022c). Cotton (*Gossypium* spp.) is a valuable commodity that is grown in various regions of the world for its fiber, fuel, and feed production. Nevertheless, crop cultivation is susceptible to diverse biotic and abiotic stresses throughout every stage of its development (Kashif et al., 2023; Nawaz et al., 2023). Despite being recognized as a crop well-suited to hot, semi-arid regions, it is observed that high temperatures (HT) have a considerable influence on cotton lint quality and the yield of its fiber (Bhardwaj et al., 2021). The influence of HT stress on cotton yield and fiber quality is determined by the severity, time period, as well as the growing stage of cotton at which the HT stress occurs (Zafar et al., 2022a). According to a previous report, it has been documented that with an increase of 1°C in the daily maximum temperature (beyond the optimal temperature range), there is a loss of 110 kg ha^-1^ in cotton yield (Singh et al., 2007). The reduction in yield is attributed to multiple factors, including a shortened boll development period (Loka et al., 2020), elevated rates of bud and boll abscission, and consequently, a decrease in the overall boll load (Schaefer et al., 2018). When cotton plants experience combined drought and HT stresses, the resulting yield loss is typically more pronounced compared to the impact of either stress alone, where the duration and intensity of the relevant stresses with regards to the growth stage determine the degree of damage (Loka et al., 2020). When the cotton crop is exposed to heat and drought stresses during its flowering and boll-forming stages, there is a potential for a significant decrease in both the quantity and quality of the fiber. This reduction renders the cotton unsuitable for subsequent processing (Hu et al., 2023). Drought and heat stress have led to substantial losses in fiber yield within the cotton industry, reaching up to 34%. Certain studies have suggested that, in accordance with the combination and conditions of applied stress, a singular of this set of stresses could potentially play a relatively predominant role (Loka et al., 2020). However, the simultaneous incident of drought and heat stress in cotton production makes it difficult to differentiate their respective impacts (Zafar et al., 2022c). Stomata, which govern the exchange of CO_2_ and water, along with the photosynthetic activity in the mesophyll, serve as the main factor influencing plant production through the regulation of photosynthesis (Nadeem et al., 2023; Nie et al., 2023). Stomatal conductance is directly affected by drought and HT stresses, leading to alterations in photosynthetic metabolism (Li et al., 2022; Gong et al., 2023). It is widely accepted fact that stomatal closure by these stresses reduces the CO_2_ uptake, which ultimately decreases the photosynthetic rate, growth and yield (Bhardwaj et al., 2021; Yin et al., 2023a). Various cotton studies have indicated that the occurrence of drought stress triggers a decrease in photosynthetic rates due to limitations imposed by both stomatal and non-stomatal factors (Cheng et al., 2022; Sultana et al., 2023; Yin et al., 2023b).

Heat and drought, like other abiotic stresses, trigger an upsurge in reactive oxygen species (ROS) production like hydrogen peroxide (H_2_O_2_), singlet oxygen, hydroxyl radicals and superoxide radical (Sekmen et al., 2014; Qin et al., 2023). Plants have developed mechanisms to counteract the production and accumulation of ROS by employing antioxidative processes (Farooq et al., 2021). These mechanisms encompass both enzymatic components, including peroxidase (POD), catalase (CAT), and superoxide dismutase (SOD), as well as non-enzymatic antioxidants such as reduced ascorbic acid, total soluble proteins, flavonoids, carotenoids, and proline (Zafar et al., 2021b). The oxidative damage incurred by cotton plants under drought and heat stress conditions is influenced by the equilibrium between ROS production and the activities of antioxidative enzymes (Manan et al., 2022). Manan et al. (2022) found that cotton produced ROS under heat stress, but the increased activities of CAT, SOD, and POD, enabling the plants to maintain the ROS scavenging process until they recovered from the stressful conditions.

As a consequence of climate change, it is anticipated that plants will encounter a higher frequency of concurrent heat and drought stress in the future (Pörtner et al., 2022; Xiong et al., 2023). The response of plant to multiple stresses is more intricate compared to a single stressor, like heat or drought. Therefore, comprehending the biochemical mechanisms that contribute to the tolerance of cotton plant to the combined stresses of heat and drought is crucial for selecting appropriate genotypes that can yield better (Rivero et al., 2022). Studies have investigated the antioxidant responses of cotton in the context of drought, heat, and salinity stresses. Nevertheless, there has been few investigations carried out on the production and detoxification of ROS by cotton plants exposed to the combined stresses of heat and drought.

This study intends to examine how the antioxidant defense systems of 12 different cotton cultivars with varying levels of drought and heat tolerance respond to the individual and combined stresses of drought and heat, specifically focusing on the formation and detoxification of ROS. The selection of these genotypes was based on field screening experiments conducted over two years at various research stations, specifically under individual heat and drought stress conditions (Chattha et al., 2017; Zafar et al., 2022a). We developed the hypothesis that the combined stress of drought and heat may elicit a distinct response in tolerant and sensitive genotypes compared to individual stresses. In contrast, when one of the stresses has a dominant effect, the response to combined stress may resemble that of a single stress. This research will provide insights into the unique responses and connections among different biochemical, physiological, agronomic and fiber quality traits of cotton to drought, heat, and combined stress. These findings can contribute to the development of strategies for enhancing cotton plants’ resilience in unstable climatic conditions.

## 2 Materials and methods

### 2.1 Plant materials

In this experiment, 12 genotypes of upland cotton (*Gossypium hirsutum* L.) were used. These genotypes were selected from previously screening experiments of 105 cotton genotypes developed by various research stations of Pakistan. The screening experiments were individually conducted for two years consecutively for drought and heat tolerance under field conditions (Chattha et al., 2017; Zafar et al., 2022a). Based on the individual screening experiments, FB-Shaheen, Eagle-2, Ghauri-1, and White Gold were identified as heat-tolerant genotypes. In contrast, FH-207, FH-329, MNH-886, and VH-291 exhibited drought tolerance. The genotypes AA-703, IR-6, KZ-191, and S-15 were found to be sensitive to the tested conditions. The seeds of these genotypes were sourced from their respective breeding stations located in Pakistan.

### 2.2 Experimental location and design

The experimental work was carried out on the research premises of FB Genetics, Four Brothers Group, Pakistan during cropping year 2021. Experimental site is located at 74˚ east longitude, and 31˚ north latitude. These genotypes were planted in 15 April 2021, under normal and stressed conditions and harvested in October 2021. A randomized complete block split-plot design with three replicates was used to conduct the experiment. Treatments were considered as main plots, and 12 cotton genotypes were assigned to the split plots. These genotypes were studied under four treatments. Plants were subjected to four treatments: a control group, drought stress (D), heat stress (H), and a combination of drought and heat stress (DH). In control conditions the total irrigation water applied to the control plot was 19.61-acre inches and additional moisture of 13.9-acre inches was in the form of precipitation. The applied irrigation water to well water condition was a total of 33.6-acre inches. Under drought stress conditions, the total irrigation water applied was 7.61-acre inches and additional moisture of 13.99-acre inches was received in the form of precipitation. The irrigation water applied in total to water-deficit condition was 21.6-acre inches. Under heat stress conditions, at flowering stage in end of July, high temperature stress was inflicted for 12 days by raising the 4-5°C temperature inside a tunnel constructed using bamboo sticks and plastic sheets. The plants within the tunnel were covered during the daytime and left uncovered during the night. To monitor the temperature inside the tunnel, a mercury thermometer was used. In the same way, under combined heat and drought stress; the total water received by well water and water deficit conditions were 33.6-acre inches and 21.6-acre inches respectively including raining water and irrigation water and an increase of 4-5°C by tunnel as described under heat stress conditions. **Figure S1** provides the recorded maximum and minimum temperature ranges observed throughout the crop growing season and **Figure S2** provides the maximum and minimum temperature inside the tunnel during heat stress conditions.

### 2.3 Data collection

#### 2.3.1 Estimation of agronomic traits

The measurement of plant height was conducted by using a measuring tape, from the first cotyledon node to the apical bud, at which point the growth ceased. The count of effective mature bolls was recorded from all the harvests, with separate records maintained for each plant. Seed cotton was collected from five guarded plants and the weight for each genotype was measured using an electronic weighing scale. The weight of each individual boll was determined by dividing the total weight of seed cotton harvested by the number of bolls collected.

#### 2.3.2 Fiber quality traits

A Testex TB510C single roller ginning machine from the USA was utilized to gin the seed cotton sample. Before ginning, the weight of the sample was recorded. The lint was then separated from the seeds, and the ginning out turn (GOT) was determined by dividing the weight of the lint by the weight of the seed cotton, expressed as a percentage. The fiber fineness, strength, and length parameters were analyzed using a high-volume instrument (HVI-900, USTER, USA).

#### 2.3.3 Quantification of biochemical and physiological traits

To collect samples for analysis, the top four fully expanded leaves were selected, and the method described by Song et al. (2014) was employed. For enzyme extraction, about 0.5 g of cotton samples were collected, and the leaves were cut using a leaf pincher. The cut leaves were then crushed and ground to obtain 1-2 ml of cold potassium phosphate buffer (pH 7.8). The resulting mixture was prepared for 5 minutes at 1,400 rpm. Afterward, the residues were removed, and the supernatant was collected for measurement of biochemical attributes using UV spectrophotometers (Evolution One Plus, Thermo Fisher Scientific) at various wavelengths, as described by (Zafar et al., 2022a). The determination of H_2_O_2_ was carried out using the Velikova protocol, as described by Velkova et al. (2000). To measure CAT, SOD, and POD activities, the protocol of (Liu et al., 2009) was adopted. To measure the protein content, the Bradford reagent method was utilized (Bradford, 1976). The Arnon method (Arnon, 1949) was used to determine carotenoids and chlorophyll a and b. To determine the ascorbic acid content, the DCIP method was employed, following the procedure described by (Davies and Masten, 1991). The quantification of total phenolic content was followed the (Ainsworth and Gillespie, 2007) method, while the measurement of flavonoid content was carried out using the (Zhishen, Mengcheng and Jianming, 1999) method. For leaf relative water contents, the method described by Silveira et al. (2009) was used to collect leaf samples for determining relative water content (RWC), with a minor modification of (Weatherley, 1950) method. The method of (Cakmak and Horst, 1991) was used to determine the MDA content in cotton leaves. Stomatal conductance (m mol m^-2^sec^-1^) was assessed at three different reproductive stages of the cotton crop using a portable infrared gas analyzer (LCi Analyzer with Broad Head, Part Number LCi-002/B and Serial Number 32455). It was measured on the fully expanded youngest leaves from 10:00 to 12:00 h.

### 2.4 Statistical analysis

Data were subject to ANOVA following split-plot arrangements. Then bar plot were drawn through “ggplot2” package in R software. Furthermore, heatmap analyses were also done through R using the package “heatmap”.

## 3 Results

Significant variations in mean square values for all the studied traits were observed among genotypes and genotype × treatment interactions, as indicated by the analysis of variance (ANOVA) results (p-values at p ≤ 0.01 and p ≤ 0.05) (**Table 1**). The biochemical, physiological, agronomic, and fiber quality traits of all cotton genotypes were significantly affected by both drought stress, heat stress, and the combined drought and heat stress. The pairwise comparisons among genotypes for all 25 traits were conducted for all four treatments (control, drought, heat and combined stress. The whole analyses are provided as **Supplementary Material (S-1)**.

**Table 1:** ANOVA split plot design for different agronomic, physiological, biochemical and fiber quality traits under control, individual (heat and drought) and combined stress conditions.

SOV	Replications	Treatments	Error A	Genotypes	Treatments × Genotypes	Error B
ASA	478.21	5199.92**	201.1	482.04**	323.04**	4684.7
BW	0.036	11.225**	0.931	1.523**	0.97**	11.039
CAR	0.004	2.076**	0.007	0.25**	0.065**	0.566
CAT	70.21	1370.07**	52.9	329.82**	59.78**	828.8
Chla	0.131	1.221**	0.02	0.665**	0.049*	2.595
Chlb	0.059	0.054**	0.006	0.068**	0.003*	0.187
FF	0.564	17.417**	0.526	5.039**	1.601**	23.113
FL	19.298	241.726**	8.830	29.178**	10.726**	167.45
FLV	5500.36	1584.46*	1102	7332.37**	215.5*	56865
FS	8.88	201.199**	6.93	45.913**	11.1**	284.88
GOT	28.099	196.298**	19.95	59.884**	13.47**	513.81
H_2_O_2_	0.027	0.137**	0.004	0.039**	0.009**	0.297
MDA	0.556	0.762**	0.152	0.341**	0.127*	6.088
NBP	8.507	100.04**	17.931	19.217**	5.98**	164.389
PH	87.7	13670.3**	158.7	238.7**	68.1**	1755.3
POD	3.076	35.479**	2.26	40.464**	7.914**	123.863
Pn	0.393	109.325**	1.24	298.95**	7.44**	12.88
Proline	0.01	2.396**	0.049	0.157**	0.083**	0.22
RWC	279.09	2130.51**	17.8	339.58**	61.58**	1946.7
SCY	4.934	484.832**	81.71	90.431**	2.685*	464.05
SOD	0.693	179.45**	0.4	6.685**	14.54**	92.77
SC	0.003	0.239**	0.003	0.16**	0.014**	0.061
TPC	1.539	47.38**	1.13	9.27**	1.87**	18.836
TR	0.424	54.44**	0.47	39.229**	1.684**	24.641
TSP	732.1	37980.2**	245	95194.1**	3503.4**	3732

* Significant at the 0.05 level.

** Significant at the 0.01 level.

Ascorbic Acid, (ASA); Boll Weight, (BW); Carotenoid Contents, (CAR); Catalase, (CAT); Chlorophyll a, (Chla); Chlorophyll b, (Chlb); Fiber Fineness, (FF); Fiber Length, (FL); Flavonoid (FLV); Fiber Strength, (FS); Ginning Out Turn percentage, (GOT%); Hydrogen Peroxide, (H2O2); Malondialdehyde, (MDA); Number of Bolls per Plant, (NBP); Plant Height, (PH); Peroxidase, (POD); Photosynthetic rate, (Pn); Superoxide Dismutase, (SOD); Relative Water Content, (RWC); Seed Cotton Yield, (SCY); Transpiration Rate, (TR); Stomatal Conductance, (SC); Total Phenolic Contents, (TPC); Total Soluble Protein, (TSP).

### 3.1 Agronomic traits

**Figure 1:**
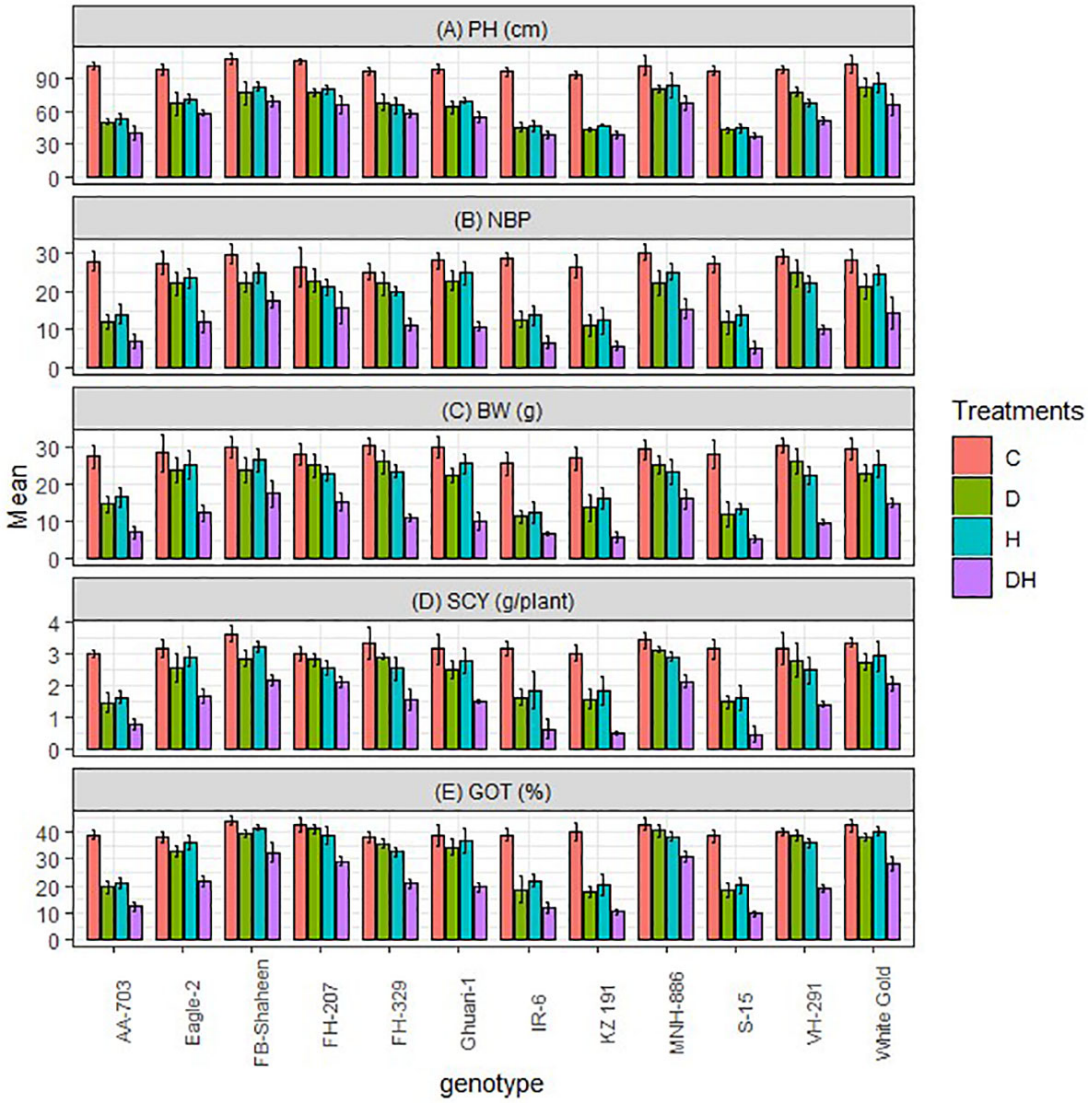
Effects of normal conditions (C), heat stress (H), drought stress (D) and combined stress (DH) conditions on agronomic traits; **(A)** Plant height, **(B)** Number of bolls per plants, **(C)** Boll weight, **(D)** Seed cotton yield, **(E)** ginning out turn% of studied cotton genotypes.

The growth and agronomic characteristics of studied genotypes were adversely influenced by heat stress (H) and drought (D) conditions, whether experienced separately or in combination (H × D). When evaluating each stressor independently, it was observed that the combined heat and drought stress had a more pronounced effect on plant height, boll number, boll weight, ginning out turn percentage (GOT %), and seed cotton yield across all genotypes (**Figure 1**). During each individual drought and heat stress, FB-Shaheen, Eagle-2, FH-207, FH-329, Ghauri-1, MNH-886, VH-291, and White Gold exhibited superior growth and yield performance in comparison to AA-703, IR-6, KZ-191, and S-15. Whereas under combined stress conditions only FB-Shaheen, FH-207, MNH-886, and White Gold were superior for PH, TNB, BW, SCY, and GOT% compared to all other genotypes (**Figure 1**). Interestingly, other tolerant genotypes could not perform well under combined stress environment for yield related traits. Under combined and single stress conditions, genotypes AA-703, IR-6, KZ-191, and S-15 exhibited significant reductions in yield parameters. These findings indicate their vulnerability and susceptibility to stress (**Figure 1**).

### 3.2 Fiber quality traits

**Figure 2:**
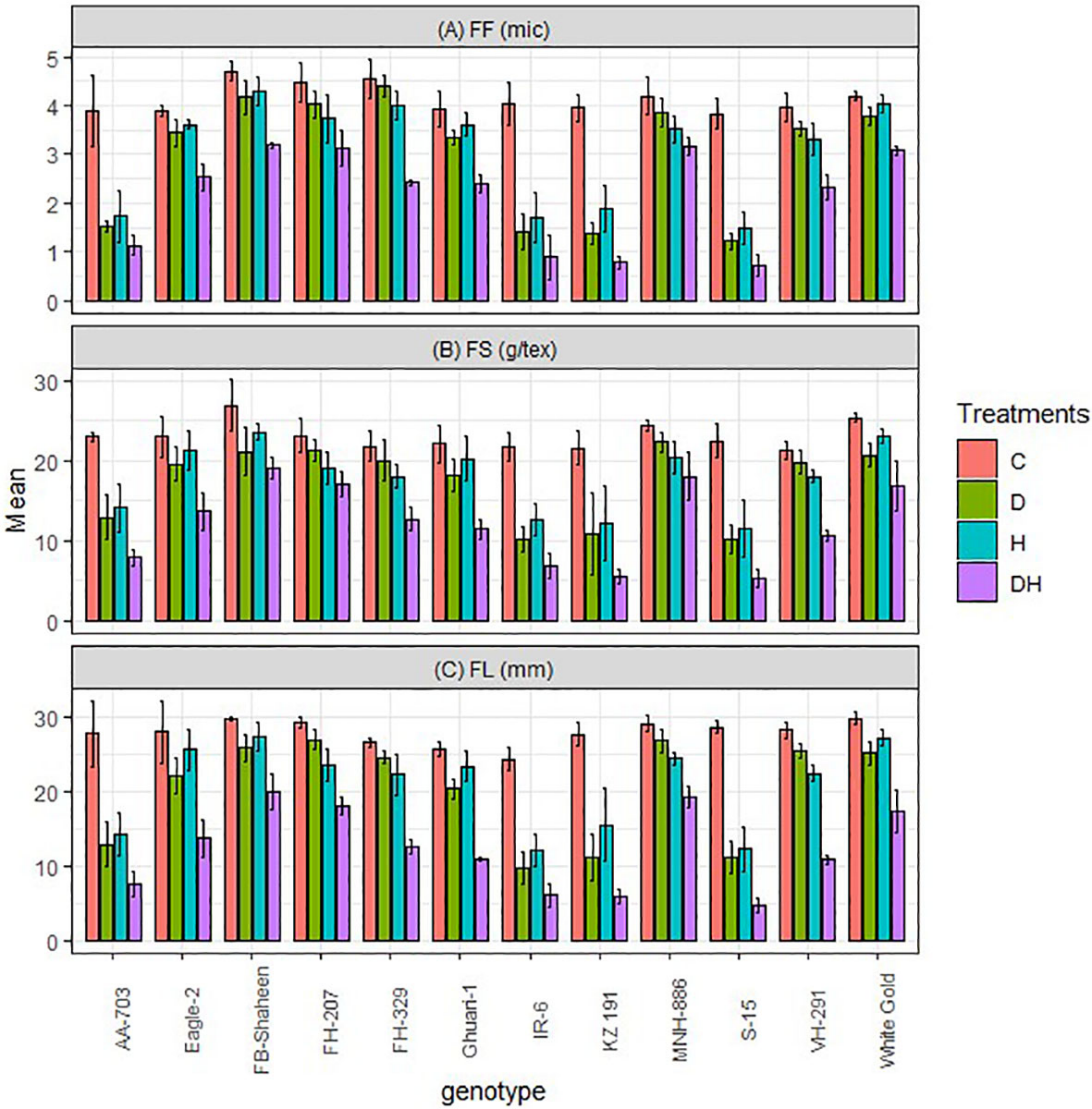
Effects of normal conditions (C), heat stress (H), drought stress (D) and combined stress (DH) conditions on fiber quality traits; **(A)** Fiber fineness, **(B)** Fiber strength, and **(C)** Fiber length of studied cotton genotypes.

The study demonstrated that both normal conditions and stressors such as drought, heat, and combined stress had significant impacts on fiber quality traits, including fiber fineness (FF), fiber strength (FS), and fiber length (FL) (**Figure 2**). However, the specific effects varied among different cotton genotypes. The study findings showed that MNH-886, FB-Shaheen, FH-207 and White Gold exhibited the best performance under both combined, and individual heat and drought stress, with the highest fiber length, strength, and fineness. In contrast, AA-703, IR-6, KZ191, and S-15 demonstrated poor performance for these fiber quality traits under all stress conditions. Additionally, Eagle-2, FH-329, Ghauri-1, and VH-291 exhibited relatively lower susceptibility to individual heat and drought stress compared to combined stress (**Figure 2**). These genotypes displayed moderate performance in terms of fiber length, strength, and fineness under combined stress conditions.

### 3.3 Physiological traits

**Figure 3:**
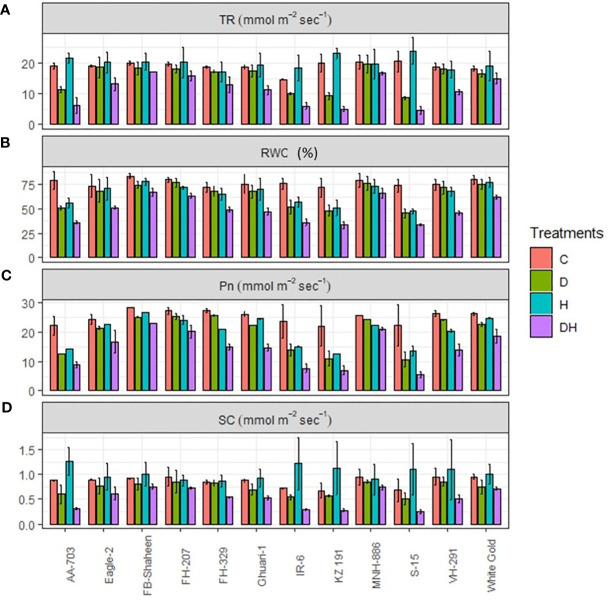
Effects of normal conditions (C), heat stress (H), drought stress (D) and combined stress (DH) conditions on physiological traits; **(A)** Transpiration rate, **(B)** Relative water contents, **(C)** Photosynthetic rate, and **(D)** Stomatal conductance of studied cotton genotypes.

Under combined stress conditions, all genotypes experienced a notable decrease in their transpiration rate (TR), photosynthetic rate (Pn), relative water content (RWC) and stomatal conductance (**Figure 3**). However, the response of different genotypes varied for transpiration rate and stomatal conductance under different individual stress treatments, with drought and heat stress were being less severe compared to combined stress. Most of the genotypes revealed higher transpiration and stomatal conductance under individual heat stress conditions. During heat stress conditions, genotypes AA-703, Eagle-2, FB-Shaheen, FH-329, Ghauri-1, FH-207, KZ191, IR-6, S-15, VH-291, and White Gold demonstrated elevated transpiration rate and stomatal conductance compared to the control conditions (**Figure 3**). MNH-886, FH-207, White Gold, and FB-Shaheen maintained their stomatal conductance and transpiration rate under individual stress treatments and showed the least decrease under combine stress environment. Conversely, genotypes such as AA-703, KZ191, IR-6, and S-15 experienced the highest reduction in stomatal conductance and transpiration rate under combined stress. Stress-sensitive genotypes, such as IR-6, AA-703, KZ 191, and S-15, showed lower Pn and RWC under drought and heat stress conditions, while stress-tolerant genotypes, such as FB-Shaheen, Eagle-2, FH-207, FH-329, Ghauri-1, MNH-886, VH-291 and White Gold showed the least decrease under both individual stresses (**Figure 3**). It is noteworthy that MNH-886, FH-207, FB-Shaheen, and White Gold displayed superior performance regarding stomatal conductance, Pn, RWC and transpiration rate, under combined stress conditions. In contrast, the remaining genotypes exhibited significant effects of combined stress on photosynthetic rate (Pn), relative water content (RWC), transpiration rate, and stomatal conductance when compared to individual stress conditions.

### 3.4 Biochemical traits

#### 3.4.1 Peroxidase (POD), Catalase (CAT), and Superoxidase (SOD)

**Figure 4:**
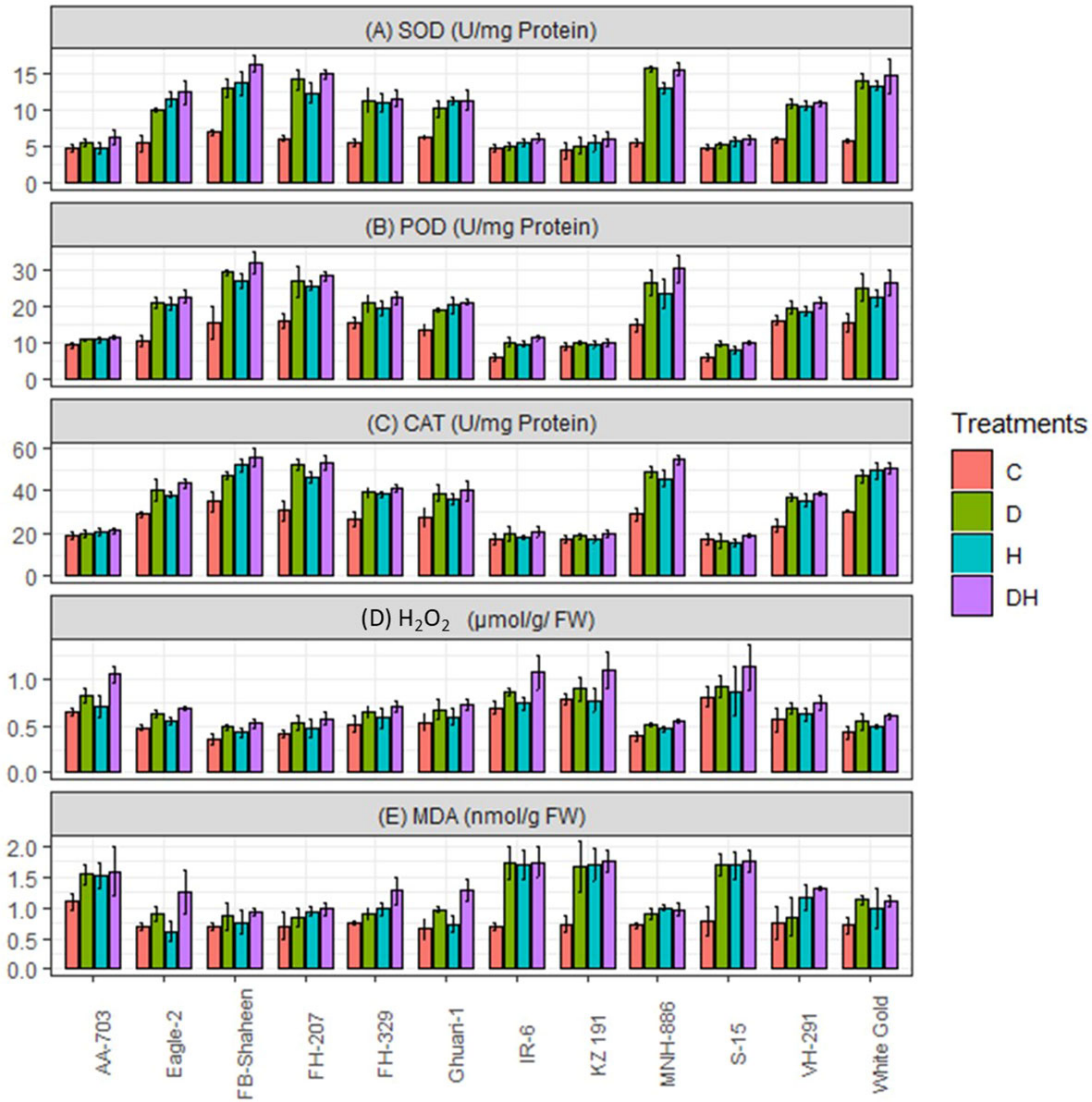
Effects of normal conditions (C), heat stress (H), drought stress (D) and combined stress (DH) conditions on physiological traits; **(A)** Superoxidase dismutase, **(B)** Peroxidase, **(C)** Catalase, **(D)** Hydrogen peroxide, and **(E)** Malondialdehyde of studied cotton genotypes.

The studied genotypes were subjected to individual and combined heat and drought stress treatments, and the activities of peroxidase (POD), catalase (CAT), and superoxidase (SOD) were assessed. All genotypes exhibited a significant increase in the activities of these antioxidant enzymes in response to both individual stresses (**Figure 4**). Notably, the tolerant genotypes (FB-Shaheen, Eagle-2, FH-207, FH-329, Ghauri-1, MNH-886, VH-291 and White Gold) exhibited the highest increase in antioxidant enzyme activities. Conversely, susceptible genotypes (AA-703, KZ 191, IR-6, and S-15) showed the least increase in these activities under both individual stress treatments (**Figure 4**). It is worth emphasizing that these susceptible genotypes experienced more pronounced negative impacts on enzymatic activities when both drought and heat stress applied simultaneously as compared to single stress. Conversely, tolerant genotypes (MNH-886, FH-207, White Gold, and FB-Shaheen) displayed greater increases in enzymatic activities under the combined treatment compared to individual stress applications (**Figure 4**). Compared to above mentioned tolerant genotypes; FH-329, VH-291, Ghuari-1, and Eagle-2 exhibited less increase in POD, CAT and SOD activities under combined stress conditions (**Figure 4**).

#### 3.4.2 Reactive oxygen species

Under all stress conditions, cotton genotypes exhibited a marked increase in the levels of reactive oxygen species (ROS). Nevertheless, the detrimental effect of combined stress was more severe in comparison to the individual stress factors. In contrast to the control, all genotypes experienced notable elevations in the levels of H_2_O_2_ and malondialdehyde (MDA) under the influence of drought stress, heat stress, and the combined stress of drought and heat. Nonetheless, the greater oxidative damage was observed in (AA-703, KZ 191, IR-6, and S-15) suggests that this cultivar exhibits lower tolerance to heat and drought stresses compared to (FB-Shaheen, Eagle-2, FH-207, FH-329, Ghauri-1, MNH-886, VH-291 and White Gold) (**Figure 4**). Under combined stress conditions the genotypes MNH-886, FH-207, FB-Shaheen, and White Gold revealed less accumulation of ROS. On the other hand, the remaining genotypes exhibited greater accumulation of H_2_O_2_ and MDA under combined stress conditions. Specifically, genotypes AA-703, KZ 191, IR-6, and S-15 demonstrated a more significant increase in H_2_O_2_ and MDA levels across all stress conditions (**Figure 4**).

#### 3.4.3 Ascorbic acid, proline, total phenolic contents, and flavonoid

**Figure 5:**
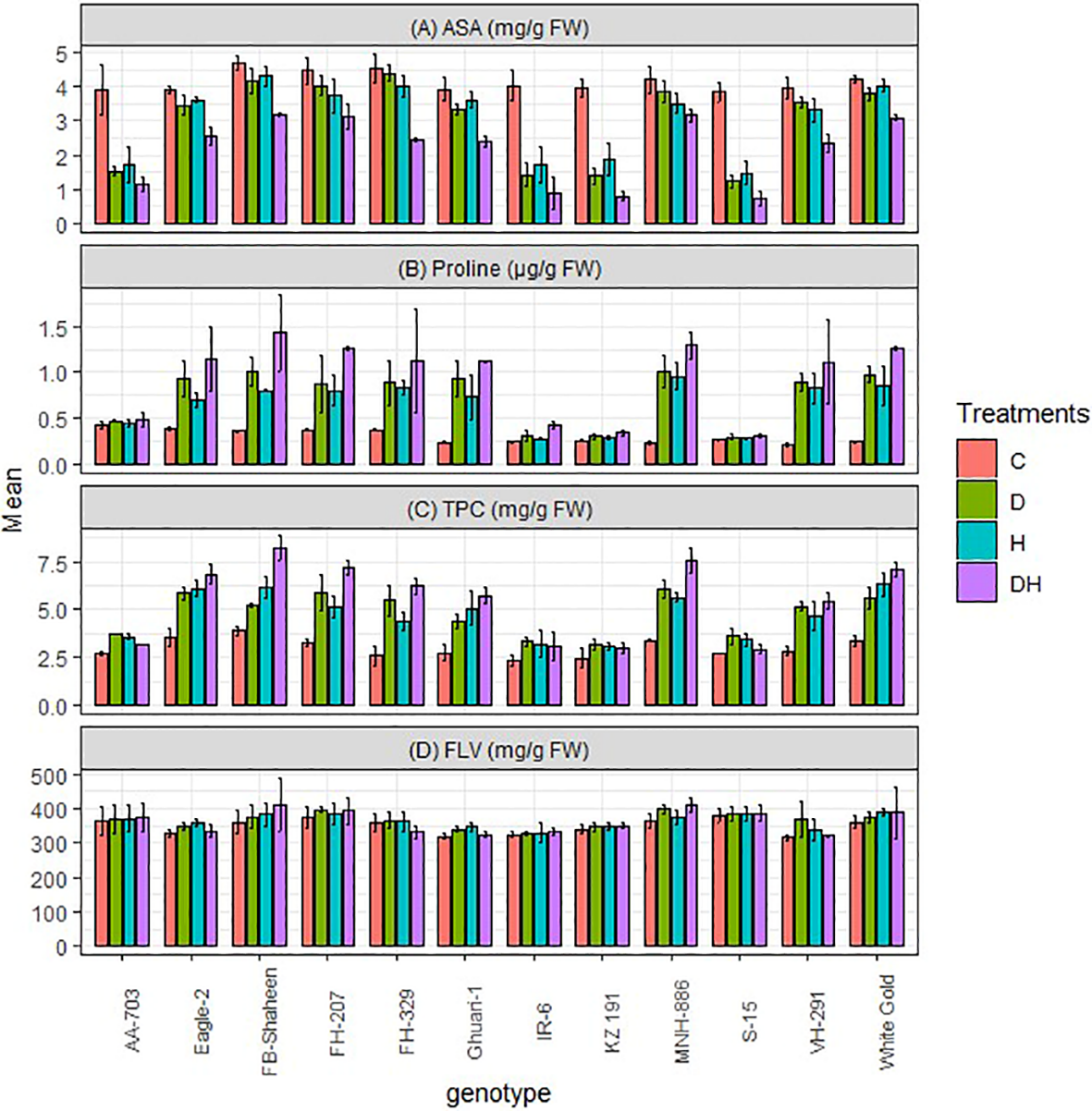
Effects of normal conditions (C), heat stress (H), drought stress (D) and combined stress (DH) conditions on **(A)** Ascorbic acid, **(B)** Proline, **(C)** Total phenolic contents and **(D)** Flavonoid contents of studied cotton genotypes.

This study revealed an augmentation in the biochemical traits of proline, total phenolic contents (TPC), flavonoid (FLV), and ascorbic acid (ASA) in cotton leaves when exposed to both individual and combined stress treatments. Under combined stress conditions, the levels of these traits were almost twice in (FB-Shaheen, FH-207, MNH-886, and White Gold) that of the control plants (**Figure 5**). Moreover, individual stress treatments resulted in a significant increase in these biochemical traits in these genotypes (FB-Shaheen, Eagle-2, FH-207, FH-329, Ghauri-1, MNH-886, VH-291 and White Gold). Remarkably, the tolerant genotypes (FB-Shaheen, FH-207, MNH-886, and White Gold) exhibited the highest concentrations of proline, total phenolic contents (TPC), flavonoid, and ascorbic acid under the combined stress conditions (**Figure 5**). Conversely, sensitive genotypes such as AA-703, S-15, KZ 191, and IR-6 performed poor for proline, TPC, flavonoid, and ascorbic acid under both individual and stress combination treatments (**Figure 5**).

#### 3.4.4 Chlorophyll a, b, total soluble protein and carotenoid contents

**Figure 6:**
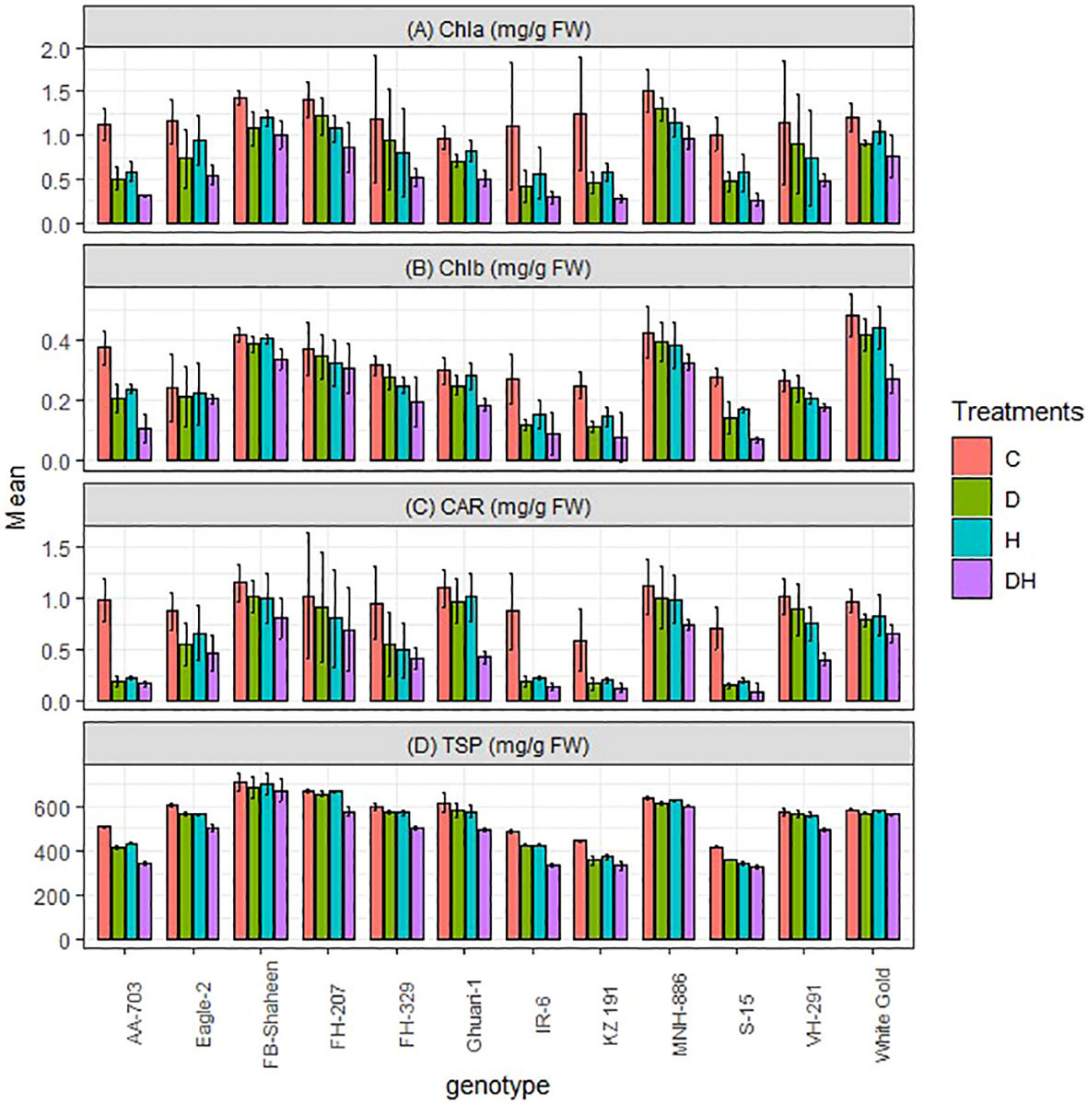
Effects of normal conditions (C), heat stress (H), drought stress (D) and combined stress (DH) conditions on **(A)** Chlorophyll a, **(B)** Chlorophyll b, **(C)** carotenoid contents, **(D)** Total soluble protein and of studied cotton genotypes.

In contrast to the previously mentioned biochemical traits, chlorophyll a (Chla), chlorophyll b (Chlb), total soluble proteins (TSP), and carotenoid (CAR) exhibited a statistically significant decrease in all genotypes when subjected to both individual and combined applications of drought and heat stress, as compared to the control treatment (**Figure 6**). The combined treatment resulted in a more pronounced decrease, compared to individual applications of drought and heat stress in susceptible genotypes. However, tolerant genotypes (FB-Shaheen, FH-207, MNH-886, and White Gold) displayed a lesser decrease in these traits. The susceptible genotypes, namely AA-703, S-15, KZ 191, and IR-6, showed the highest reduction in pigments carotenoid, chlorophyll a and chlorophyll b levels (**Figure 6**). This was followed by FH-329, VH-291, Ghauri-1, and Eagle-2, while the tolerant genotypes (FB-Shaheen, FH-207, MNH-886, and White Gold) showed the least reduction in these pigment levels. Notably, the total soluble protein was not significantly affected in the aforementioned tolerant genotypes compared to the susceptible genotypes mentioned earlier (**Figure 6**).

#### 3.4.5 Correlation analysis

**Figure 7:**
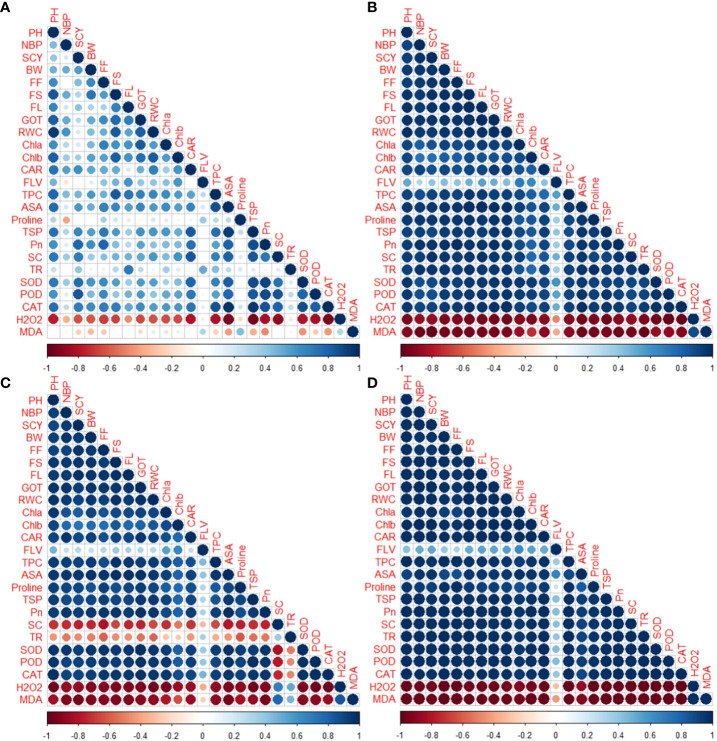
Correlation between different agronomic, physio-chemical, and fibre-related traits in upland cotton genotypes under control **(A)**, drought **(B)**, heat **(C)** and combined **(D)** stress conditions.

Correlation analysis was conducted on studied genotypes, examining their physiological, biochemical, agronomic and fiber quality characteristics under normal and stressed conditions (**Figure 7**). Positive and significant correlations were noted between SCY and several other traits including agronomic, fiber quality traits, physiological, and biochemical traits under drought stress conditions. Conversely, the SCY exhibited a negative correlation with MDA and H_2_O_2_. The H_2_O_2_ and MDA also revealed negative association with PH, NBP, BW, GOT%, SCY, FL, FS, FF, BW, Car, chlorophyll contents, CAT, proline contents, TSP, TPC, SC, Pn, ASA, SOD, POD, FLV and RWC under drought stress conditions (**Figure 7**). Under heat stress environments, stomatal conductance and transpiration rate revealed significant positive correlation with ROS (H_2_O_2_ and MDA). Whereas TR and SC showed negative association with antioxidant activities, yield and fiber quality traits. Both H_2_O_2_ and MDA were also negatively associated with all other biochemical, physiological, fiber quality and yield contributing characters under heat stress conditions. Under combined heat and drought stress conditions, FLV exhibited lower positive relationship with other antioxidants, yield and fiber quality traits. Whereas PH, NBP, BW, GOT%, SCY, FL, FS, FF, BW, Car, chlorophyll contents, CAT, proline contents, TSP, TPC, SC, Pn, ASA, SOD, POD, FLV and RWC showed significant positive correlation among themselves under combined stress conditions (**Figure 7**). Both H_2_O_2_ and MDA revealed significant and negative association with biochemical, physiological, fiber quality and agronomic traits under combined drought and heat stress conditions (**Figure 7**).

#### 3.4.6 Heat mapping agro-physiological, biochemical, and fiber quality traits of cotton genotypes under individual and combined stress conditions

**Figure 8:**
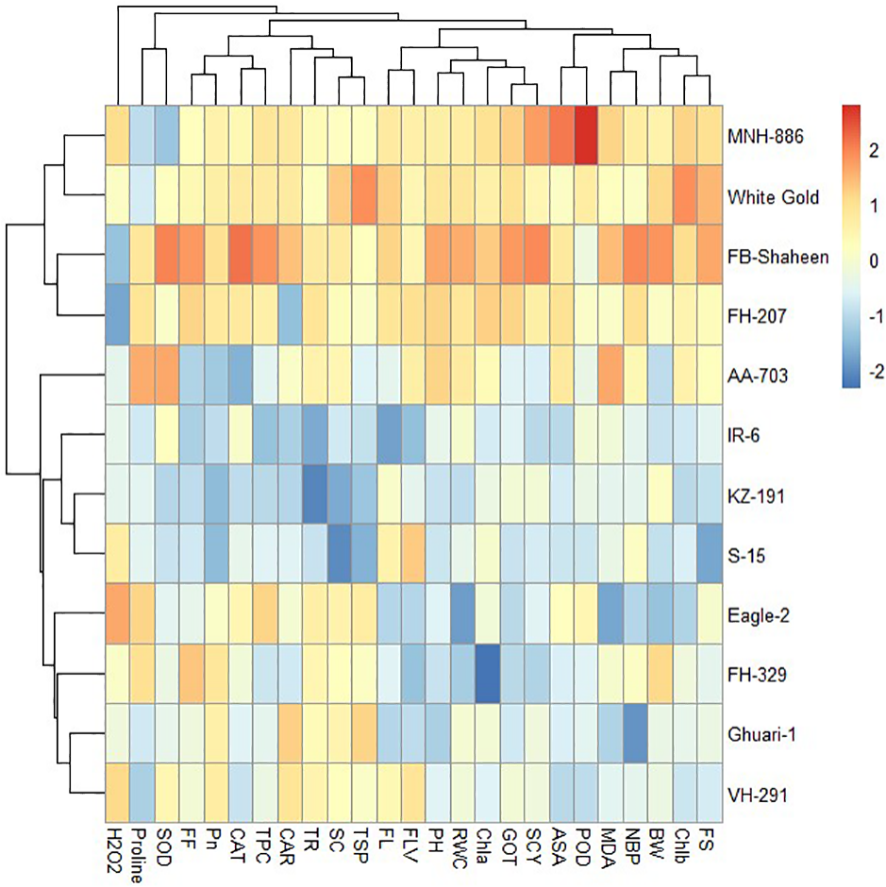
Heat map analysis of various traits under normal conditions.

**Figure 9:**
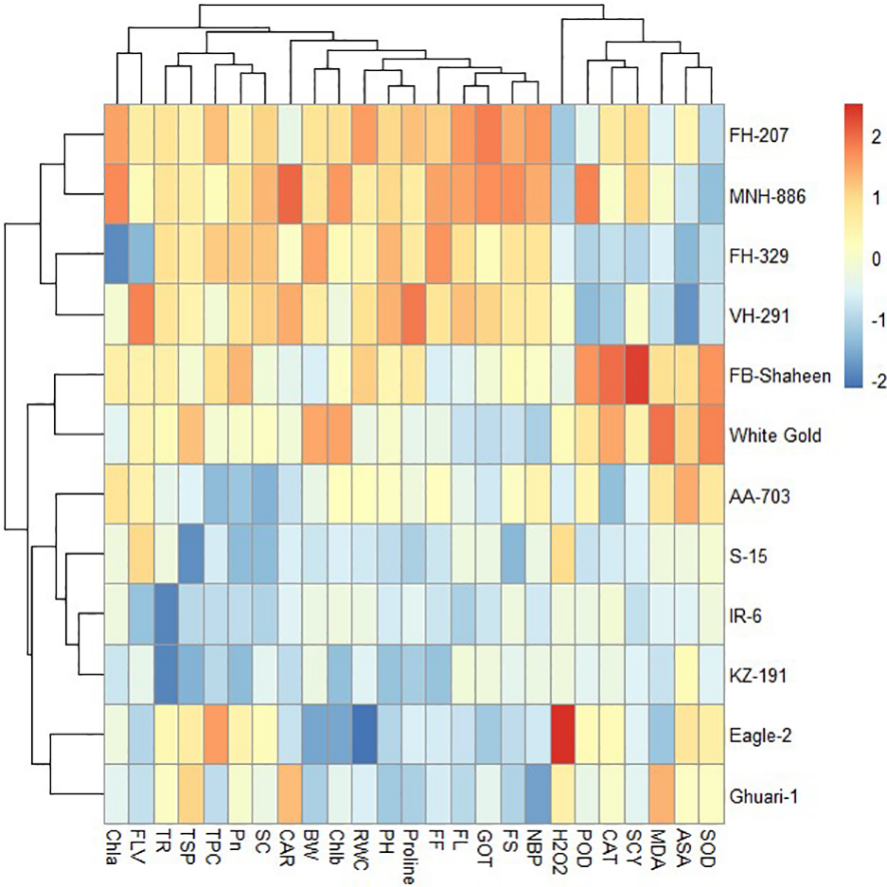
Heat map analysis of various traits under drought stress.

The heat map illustrates the relative performance of different genotypes under normal, individual and combined stress conditions. The heat maps clustered the cotton genotypes on the basis of mean into different groups. Under control conditions the genotypes were divided into three clusters based on performance for the studied traits (**Figure 8**). Under drought stress conditions, the 12 cotton genotypes were grouped into four clusters based on agronomical, biochemical, and fiber quality characteristics. Genotypes belonging to cluster-I, (FH-207, MNH-886, FH-329, and VH-291) demonstrated higher values for agronomic and antioxidant traits and lower values for MDA and H_2_O_2_, indicating their resistance to drought stress. Whereas the genotypes of cluster-II (FB-Shaheen, and White Gold) revealed moderate performance agronomic and antioxidant traits under drought stress conditions. The genotypes of cluster-III (AA-703, S-15, IR-6, and KZ-191) were highly sensitive to drought stress followed by genotypes of cluster-IV (Eagle-2, and Ghauri-1) (**Figure 9**).

**Figure 10:**
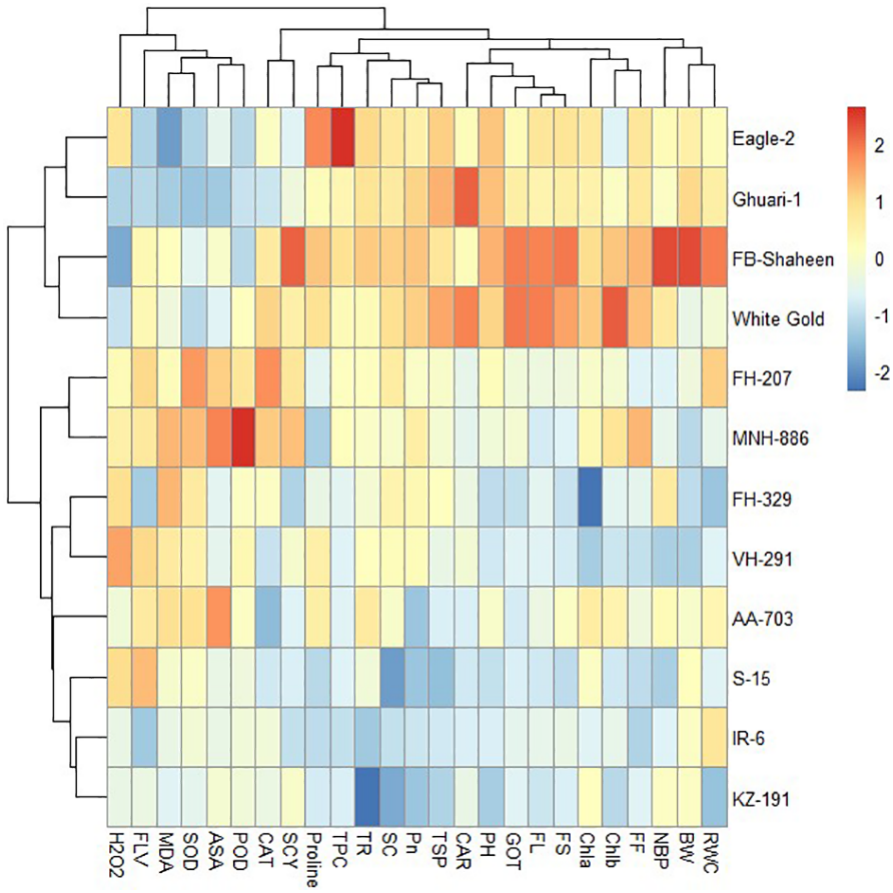
Heat map analysis of various traits under heat stress.

Under heat stress conditions, 12 cotton genotypes were categorized into three clusters based on agronomical, biochemical, and fiber quality traits. Genotypes in cluster-I (Eagle-2, FH-207, FH-329, Ghauri-1, FB-Shaheen, and White Gold) displayed higher values for agronomic and antioxidant traits, while showing lower values for MDA and H_2_O_2_, indicating their resilience to heat stress. On the other hand, genotypes in cluster-II (FH-207, MNH-886, FH-329, and VH-291) demonstrated moderate performance in agronomic and antioxidant traits under heat stress conditions. The genotypes in cluster-III (AA-703, S-15, IR-6, and KZ-191) were highly sensitive to heat stress conditions and perform poor for agronomic, biochemical, and fiber quality traits (**Figure 10**).

**Figure 11:**
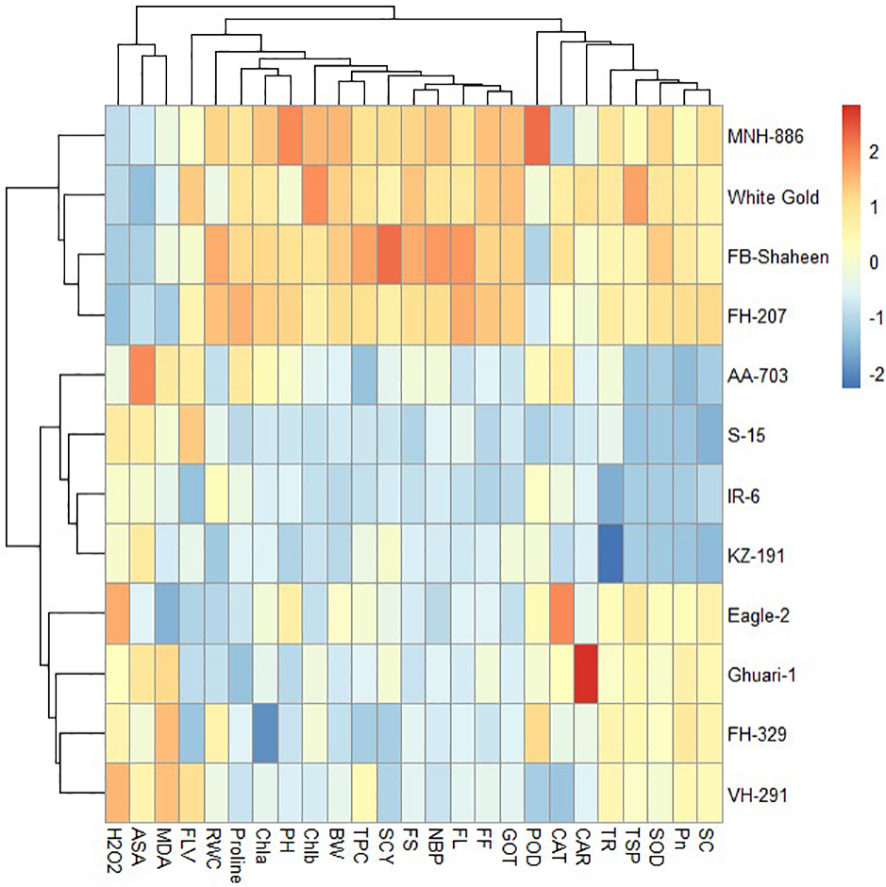
Heat map analysis of various traits under combined stress condition.

Under combined drought and heat stress conditions, the 12 cotton genotypes were classified into three clusters based on studied characteristics. Cluster-I consisted of FB-Shaheen, FH-207, MNH-886, and White Gold, which exhibited higher values for PH, TNB, BW, SCY, GOT%, POD, CAT, SOD, TPC, proline, ASA, FLV, RWC, Tr, Pn, CAR, Chla, Chlb, TSP, FS and FL and lower values for MDA and H_2_O_2_, indicating their resistance to combined stress conditions. In contrast, the genotypes of cluster-II (AA-703, IR-6, KZ-191, and S-15) demonstrated poor performance for above mentioned agronomic and antioxidant traits under combined stress conditions. The genotypes in cluster-III (Eagle-2, Ghauri-1, FH-329, and VH-291) were moderately tolerant to combined stress conditions and exhibited moderate performance for agronomic, biochemical and fiber quality traits (**Figure 11**).

## 4 Discussion

Drought and high temperatures are recognized as critical challenges that hinder cotton’s ability to achieve high yield potential (Loka et al., 2020). Climate models project a rise in surface temperature by 3–5°C in the next century, resulting in an increased occurrence of droughts and floods (Soong et al., 2020). The escalating threat of climate-related events to agriculture as well as global food security, coupled with the rapid growth of the world population, necessitates the urgent development of stress-tolerant crops (Zafar et al., 2021a). Considering the ongoing fluctuations in the climate, it has become essential to systematically expose cotton plants to different stresses (Haroon et al., 2023). This approach is crucial for identifying genotypes that are resilient to climate changes and can be effectively exploited in future breeding programs (Nawaz et al., 2023). The main objective of this study was to investigate the impacts and combined influence of heat and drought stresses on the morphological, physiological, biochemical, and agronomic traits of 12 cotton genotypes (comprising 8 tolerant and 4 susceptible genotypes). The findings demonstrated that all genotypes exhibited favorable performance under normal conditions, but both drought and heat stresses individual and combined had detrimental effects on various agronomic traits (Dabbert et al., 2017). Interestingly, the impact of these two stresses on all traits was relatively similar across the tolerant genotypes (FB-Shaheen, Eagle-2, FH-207, FH-329, Ghauri-1, MNH-886, VH-291 and White Gold) whereas sensitive genotypes (AA-703, KZ 191, IR-6, and S-15) revealed more destructive effects on agronomic traits PH, NBP, BW, SCY, and GOT%. These effects can be attributed to the accelerated maturity of cotton plants due to a shortened flowering-to-boll opening period under stressful conditions (Pettigrew, 2004), as well as a reduction in the time available for cotton boll accumulation (Xu et al., 2019). Nevertheless, the results of our research suggest that the decline in cotton yield resulting from a decrease in the number of bolls has a greater effect compared to the impact of reduced boll weight. These findings are consistent with the observations made by Hu et al. (2018) (Hu et al., 2018). Cotton yield is further constrained by water scarcity and heat stress, leading to a decrease in boll numbers (Snider et al., 2019; Zafar et al., 2022a). However, when subjected to DH, drastic effects of combined stress was observed on all cotton genotypes for agronomic characters compared to individual stress (Gao et al., 2021). Only four genotypes (FB-Shaheen, FH-207, MNH-886, and White Gold) showed higher performance for agronomic traits whereas rest of four tolerant genotypes revealed moderate performance in combined stress conditions. The sensitive genotypes showed poor performance under combined stress environmental conditions. These observations suggest that the evolving climatic conditions are expected to have a significantly negative impact on cotton genotypes (Manan et al., 2022).

The current study revealed elevated levels of H_2_O_2_ and MDA in cotton genotypes when exposed to individual drought, heat, and combined stress conditions.

The observed correlation between the extent of oxidative damage and the vulnerability of cotton genotypes to combined drought and heat stress suggests a direct relationship (Zandalinas et al., 2018). The sensitive genotypes revealed higher accumulation of H_2_O_2_ and MDA followed by moderately tolerant and tolerant genotypes (Qaseem et al., 2019). The oxidative damage caused by the accumulation of H_2_O_2_ and MDA adversely affects essential cellular components such as DNA, lipids and proteins. Consequently, this damage leads to cell death and hampers the overall growth of plants (Zafar et al., 2021b). Proline serves as an osmo-protectant in plants, and is actively involved in multiple stress signaling pathways enabling them to endure stressful conditions. When plants experience stress, they tend to accumulate higher levels of proline (Alagoz et al., 2023). The accumulation of proline is essential for combating stress as it effectively scavenges ROS from the cell, safeguarding it against ROS-induced cellular damage, while preserving the normal biological activities within the cell. This mechanism protects the cell and helps maintain the structural integrity of proteins (Alagoz et al., 2023). Our research focused on evaluating proline accumulation and revealed that sensitive genotypes exhibited reduced proline levels under stress conditions whereas the tolerant genotypes, however, (FB-Shaheen, FH-207, MNH-886, and White Gold) exhibited increased level of proline under both individual as well as combined stress conditions. Under separate and combined stress conditions, tolerant plants exhibited a significant rise in proline accumulation, consistent with the study (Raja et al., 2020). This study revealed that increased proline levels serve as an osmoprotectant in plants, safeguarding them against the combined stresses of drought and heat, as reported by (Moreno-Galván et al., 2020). RWC acts as a reliable indicator of stress resistance in plants across diverse stress conditions. Plants exhibiting decreased RWC content are typically regarded as more vulnerable to both individual and combined stress conditions, primarily due to enhanced protoplasmic permeability (Aslam et al., 2023), similarly, we also observed reduced RWC value in sensitive genotypes, indicating their susceptibility to stress. The species which are able to maintain RWC are considered as tolerant/resistant to combined stress (Zandalinas et al., 2018). Chlorophyll pigments are engaged directly in metabolic processes plants. The reduction in chlorophyll contents is directly associated with reduced growth, productivity as well as tolerance (Nawaz et al., 2023). Likewise, we also found a substantial reduction in chlorophyll content among all genotypes during individual as well as combined stress, which might be attributed to harm to the chloroplasts (Zafar et al., 2022c). In comparison to tolerant cultivars, drastic reductions in chlorophyll contents were observed in sensitive genotype under combined stress conditions. Carotenoids, known for their antioxidant properties, play a defensive role by preventing chlorophyll photo-oxidation (Mekki et al., 2015). All stress treatments have a notable impact on carotenoids contents while most substantial reduction was found under combined stress environment (Ashraf et al., 2019). Previous studies have also reported the reduction in the contents of carotenoid under several stress environments (Manan et al., 2022; Zafar et al., 2022c). Carotenoid have ability in quenching the singlet oxygen, therefore, relative levels of carotenoid can act as an indicator of tolerance (Ashraf et al., 2019; Manan et al., 2022). Our study found the decline in transpiration rate, stomatal conductance and overall photosynthetic rate while examining photosynthetic performance during combined stress (heat and drought). Notably, the combination of heat and drought stress exhibited a severe effect on photosynthesis (El Sabagh et al., 2020). Moreover, we ascertained that heat and drought stress act synergistically, simultaneously limiting photosynthesis in field conditions. Total soluble protein was reduced in all genotypes when exposed to single stress (heat and drought) as well as combined stresses. This reduction in protein content might be ascribed to a significant decline in photosynthesis (Shallan et al., 2012) or a limited availability of protein assimilates, which are crucial for protein synthesis. The convergence of drought and heat leads to an intensified negative effect on photosynthesis (Ru et al., 2022), encompassing both stomatal and non-stomatal limitation (Zhou et al., 2020). Plants have evolved protective mechanism to mitigate the harmful impact of reactive oxygen species (ROS). These mechanisms involve the synthesis of both enzymatic and non-enzymatic antioxidants. Enzymatic antioxidants like catalase peroxidase (POD), superoxide dismutase (SOD) and CAT are crucial in breaking down ROS into less harmful substances (Zafar et al., 2022b). In our experiment, we observed that the tolerant genotypes exhibited higher expression levels of ROS-scavenging enzymes, including SOD, POD, and CAT, when subjected to stress conditions. This likely contributed to their superior performance, and similar results were documented by (Zhanassova et al., 2021). Other researchers (Niu et al., 2018; Hanif et al., 2021) have also reported increased activities of catalase (CAT) and superoxide dismutase (SOD) under both individual and combined stress conditions, as observed in our current study. However, under combined stress conditions, the moderately tolerant genotypes displayed only moderate levels of SOD, POD, and CAT. In contrast, the susceptible genotypes showed minimal accumulation of these enzymes. In addition to enzymatic components, non-enzymatic components including phenols, flavonoids, and ascorbic acid also contribute to preventing the effects of oxidative stress by improving the scavenging of ROS (Yildiz-Aktas et al., 2009). Flavonoids have been found to provide photoprotection to plants against high temperatures and drought stress (De Souza Junior et al., 2023). In the present study, flavonoid contents were enhanced among tolerant genotypes when exposed to combined heat and drought stress as compared to individual heat or drought stresses. However, moderately tolerant genotypes showed a lesser extent of increase in flavonoid content. Susceptible genotypes did not exhibit any increase in flavonoid content.

Plants usually enhance the production of secondary metabolites, such as polyphenols, as a typical response to abiotic stresses. Phenolics, in particular, confer higher tolerance to plants against drought and heat stress (Ashraf et al., 2019). Similarly, phenolic contents were significantly enhanced in tolerant genotypes under combined stress as compared to the control group. Conversely, moderately tolerant genotypes exhibited a slight elevation in phenolic content compared to individual stress conditions. There is a scarcity of specific reports that discuss the synergistic impacts of drought and heat stress on the accumulation of phenols and antioxidant potential.

Under individual stress treatments, we found that non-enzymatic antioxidant ascorbic acid (ASA) was enhanced significantly in plant’s leaves. Ascorbic acid actively participates in the ascorbate-glutathione cycle, which involves APX enzymes. Moreover, under stress conditions, ensuring sufficient levels of these enzymes is critical as they serve as guardians of cells, protecting them from oxidative damage (Wu et al., 2015). The increased ASC levels found in our study align with previous findings in drought-stressed chickpea (El-Beltagi et al., 2020), sweet pepper (Khazaei and Estaji, 2020), and wheat (Gupta and Thind, 2015).

Both fiber yield and quality are affected by the simultaneous occurrence of water deficit and high temperatures. These stresses interacted and influenced the formation of fiber length. When temperatures surpassed the optimal range, fiber length reduction was observed due to a shortened period of fiber elongation. Additionally, drought disrupts the equilibrium of turgor pressure within cotton fiber cells, resulting in a reduction in fiber length (El Sabagh et al., 2020; Zafar et al., 2022a). Similarly, high temperature exacerbates the negative effects of water deficit on length of the fiber under combined conditions, with more severe drought stress leading to stronger co-effects (Lokhande and Reddy, 2014; Hu et al., 2022). However, under combined stress, the performance of these genotypes was moderate in comparison to the tolerant genotypes. In contrast, susceptible genotypes exhibited a notable decrease in fiber quality and yield-related traits.

## 5 Conclusion

The findings of this study provide valuable insights into the intricate interplay of combined heat and drought stress and their effects on cotton plants, thereby making a noteworthy contribution to our comprehension of these complex interactions. Through a comprehensive investigation of physiological, biochemical, and yield traits, we have gained valuable insights into how cotton genotypes respond to this multifaceted challenge. The results highlight pronounced differences in stress tolerance among the categorized cotton genotypes under combined stress conditions. The tolerant varieties demonstrated remarkable resilience compared to the susceptible and moderately tolerant ones, as evidenced by their ability to maintain higher yields and favorable fiber traits when subjected to the combined stress treatment. However, the accumulation of various osmolytes, enzymatic as well as non-enzymatic antioxidants, can enhance the tolerance of cotton crop under these challenging conditions. It is important to note that studies focusing solely on individual abiotic stresses do not fully capture the specific responses of cotton plants to combinations of different stresses, which can significantly impact crop performance in field conditions. By investigating the alterations and interconnections among the physiological and biochemical responses of cotton genotypes to drought, heat, and combined stress, this study aims to make a substantial contribution to the identification and breeding of resilient cotton cultivars capable of withstanding both single and combined stress conditions. This research sets the stage for future studies that explore additional traits, such as genetic markers associated with stress tolerance, and investigate the long-term impacts of combined stress on cotton yield and its quality.

## Data availability statement

The original contributions presented in the study are included in the article/**Supplementary Material**. Further inquiries can be directed to the corresponding authors.

## Author contributions

MZ: Conceptualization, Investigation, Supervision, Writing – original draft, Writing – review & editing. WC: Conceptualization, Visualization, Writing – review & editing. AK: Project administration, Supervision, Writing – review & editing. SZ: Data curation, Writing – review & editing. MS: Formal Analysis, Visualization, Writing – review & editing. HS: Supervision, Validation, Visualization, Writing – review & editing. AA: Formal Analysis, Software, Validation, Visualization, Writing – review & editing. AI: Visualization, Validation, Writing – review & editing. ZA: Validation, Visualization, Writing – review & editing. FQ: Funding acquisition, Supervision, Writing – review & editing. AS: Methodology, Validation, Writing – review & editing. MFS: Funding acquisition, Supervision, Validation, Writing – review & editing. DW: Formal Analysis, Software, Writing – review & editing. AP: Conceptualization, Writing – review & editing. AR: Investigation, Methodology, Writing – review & editing. JX: Funding acquisition, Supervision, Visualization, Writing – review & editing.

## Supplementary material

Supplementary Figure 1 Weather data during the crop seasons of 2021.

Supplementary Figure 2 Temperature recorded in the tunnel during experiments.

Supplementary Table 1 Pairwise comparison of all genotypes for studied traits under normal and stressed conditions.
