# Supplementary figures and images for: Drought and heat stress on cotton genotypes suggested agro-physiological and biochemical features for climate resilience
Source: Front Plant Sci. 2023 Oct 30;14:1265700. doi: 10.3389/fpls.2023.1265700 (PMC10643170; doi:10.3389/fpls.2023.1265700)

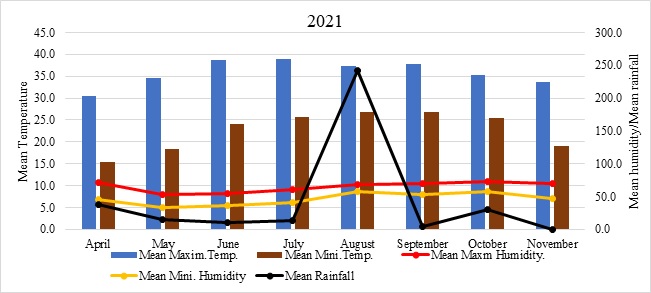

Supplement: Supplementary Figure 1 — Weather data during the crop seasons of 2021. [file Image_1.jpg]

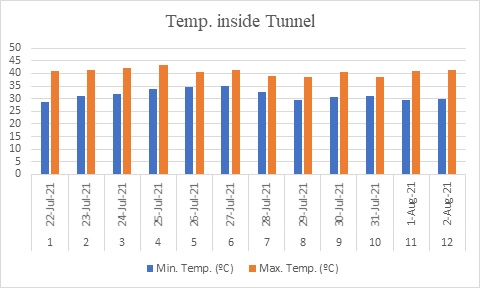

Supplement: Supplementary Figure 2 — Temperature recorded in the tunnel during experiments. [file Image_2.jpg]
